# Role of the right temporoparietal junction in intergroup bias in trust decisions

**DOI:** 10.1002/hbm.24903

**Published:** 2019-12-19

**Authors:** Junya Fujino, Shisei Tei, Takashi Itahashi, Yuta Y. Aoki, Haruhisa Ohta, Manabu Kubota, Ryu‐ichiro Hashimoto, Hidehiko Takahashi, Nobumasa Kato, Motoaki Nakamura

**Affiliations:** ^1^ Medical Institute of Developmental Disabilities Research Showa University Setagaya‐ku Tokyo Japan; ^2^ Department of Psychiatry, Graduate School of Medicine Kyoto University Sakyo‐ku Kyoto Japan; ^3^ Institute of Applied Brain Sciences Waseda University Tokorozawa Saitama Japan; ^4^ School of Human and Social Sciences Tokyo International University Kawagoe Saitama Japan; ^5^ Department of Psychiatry, School of Medicine Showa University Setagaya‐ku Tokyo Japan; ^6^ Department of Functional Brain Imaging National Institute of Radiological Sciences, National Institutes for Quantum and Radiological Science and Technology Inage‐ku Chiba Japan; ^7^ Department of Language Sciences, Graduate School of Humanities Tokyo Metropolitan University Hachioji‐shi Tokyo Japan; ^8^ Department of Psychiatry and Behavioral Sciences, Graduate School of Medical and Dental Sciences Tokyo Medical and Dental University Bunkyo‐ku Tokyo Japan; ^9^ Kanagawa Psychiatric Center Yokohama Kanagawa Japan

**Keywords:** decision‐making, intergroup bias, repetitive transcranial magnetic stimulation, temporoparietal junction

## Abstract

Intergroup bias, which is the tendency to behave more positively toward an in‐group member than toward an out‐group member, is pervasive in real life. In particular, intergroup bias in trust decisions substantially influences multiple areas of life and thus better understanding of this tendency can provide significant insights into human social behavior. Although previous functional magnetic resonance imaging studies showed the involvement of the right temporoparietal junction (TPJ) in intergroup trust bias, a causal relationship between the two has rarely been explored. By combining repetitive transcranial magnetic stimulation and a newly developed trust game task, we investigated the causal role of the right TPJ in intergroup bias in trust decisions. In the trust game task, the counterpart's group membership (in‐group or out‐group) and reciprocity were manipulated. We applied either neuronavigated inhibitory continuous theta burst stimulation (cTBS) or sham stimulation over the right TPJ before performing the trust game task in healthy volunteers. After the sham stimulation, the participants' degrees of investments with in‐group members were significantly higher than those with out‐group members. However, after cTBS to the right TPJ, this difference was not observed. The current results extend previous findings by showing that the causal roles of the right TPJ can be observed in intergroup bias in trust decisions. Our findings add to our understanding of the mechanisms of human social behavior.

## 1 INTRODUCTION

Groups are a pervasive feature of our social lives. We interact with people who share common group identities, such as nations, religions, and political parties, and we find ourselves interacting with others who belong to different groups (Akerlof & Kranton, 2000; De Dreu et al., 2010; Fiske, 2002; Levine, Prosser, Evans, & Reicher, 2005; Tajfel, Billig, Bundy, & Flament, 1971). Intergroup bias is the tendency to behave more positively toward an in‐group member than toward an out‐group member (Balliet, Wu, & De Dreu, 2014; Baumgartner, Nash, Hill, & Knoch, 2015; Baumgartner, Schiller, Hill, & Knoch, 2013; Bernhard, Fischbacher, & Fehr, 2006; Chen & Li, 2009; De Dreu & Kret, 2016; Ellemers, 2012). For example, people often evaluate in‐group members more positively than out‐group members (Ahmed, 2007; Hewstone, Rubin, & Willis, 2002; Worchel, Rothgerber, Day, Hart, & Butemeyer, 1998); they also tend to reward in‐group members more than out‐group members (Balliet et al., 2014; Cikara & Van Bavel, 2014). Intergroup bias is highly prevalent in real life and thus has been documented in various disciplines, including psychology (Brewer, 1999; Halevy, Weisel, & Bornstein, 2012), economics (Ben‐Ner, McCall, Stephane, & Wang, 2009; Goette, Huffman, & Meier, 2006), politics (Falk, Spunt, & Lieberman, 2012; Rand et al., 2009), and neuroscience (Baumgartner, Götte, Gügler, & Fehr, 2012; Baumgartner, Schiller, Hill, & Knoch, 2013).

Trust is essential for initiating, establishing, and maintaining social relationships (Balliet & Van Lange, 2013; Bellucci, Chernyak, Goodyear, Eickhoff, & Krueger, 2017; King‐Casas et al., 2005; Kosfeld, Heinrichs, Zak, Fischbacher, & Fehr, 2005; Krueger et al., 2007; McAdams, Lohrenz, & Montague, 2015) and facilitates the flourishing of groups, organizations, and nations (Balliet & Van Lange, 2013; Delgado, Frank, & Phelps, 2005; Labonne & Chase, 2010; Riegelsberger, Sasse, & McCarthy, 2005). Trust results in greater relationship commitment and satisfaction (Campbell, Simpson, Boldry, & Rubin, 2010; Van't Wout & Sanfey, 2008), whereas broken trust may mark the demise of social relations (Balliet & Van Lange, 2013; Tzieropoulos, 2013). Previous studies using a variety of self‐report, implicit, and behavioral measures reveal that people typically trust in‐group members more than out‐group members (Balliet et al., 2014; De Dreu & Kret, 2016; Romano, Balliet, Yamagishi, & Liu, 2017). Such intergroup trust bias in its extreme can foster intergroup conflict: it creates feelings of deprivation and resentment in out‐groups, the members of which may respond with hostility toward the distrusting in‐group (Balliet et al., 2014; Baumgartner, Schiller, Rieskamp, Gianotti, & Knoch, 2013). Subsequently, it may lead to severe outcomes, such as excessive competition, discrimination, and violent protest (De Dreu & Kret, 2016; Romano et al., 2017). However, this tendency to extend trust toward in‐group members not only improves group functioning but also enables the individual to fit into a group (Balliet et al., 2014; Baumgartner, Schiller, Rieskamp, et al., 2013). The tendency brings a wide variety of advantages, such as safety and security the group provides against outside threats, inclusion in potentially beneficial exchanges with others, and social support (Balliet et al., 2014; Baumgartner, Schiller, Rieskamp, et al., 2013). Conversely, impairments in such group psychology undermine social inclusion and fitting in (Balliet et al., 2014; De Dreu & Kret, 2016). Individuals who chronically suffer from these impairments, including those diagnosed with autism spectrum disorder (ASD), schizophrenia, and borderline personality disorder, risk a lack of social support and have a reduced well‐being (De Dreu & Kret, 2016; King‐Casas et al., 2008; Tei et al., 2019). Thus, an improved understanding of intergroup trust bias can provide significant insights into social cognitive functioning and its impairments in psychiatric disorders.

To date, several previous functional magnetic resonance imaging (fMRI) studies have investigated the neural mechanisms of intergroup bias and found a pivotal role of the temporoparietal junction (TPJ), especially in two decision situations: punishing behavior and trust decisions. For example, a previous study using a third‐party punishment task found increased activity in the TPJ and dorsomedial prefrontal cortex when third parties were confronted with defecting in‐group members compared with defecting out‐group members (Baumgartner et al., 2012). As for the intergroup bias in trust decisions, Hughes, Ambady, and Zaki (2017) showed that the TPJ, dorsal anterior cingulate cortex and lateral prefrontal cortex were functionally coupled with the striatum during intergroup trust decisions. In addition, a recent study reported that the TPJ plays a key role in trust behavior when playing with partners of the same or different political identities (Wu et al., 2018). In particular, the authors revealed that the neural activation of the TPJ is associated with unexpected negative outcomes in in‐group members (Wu et al., 2018).

Although fMRI is a promising technique to investigate the neural correlates of the task performance, it does not permit causal inferences about the effect of brain processes on human behavior because of the cross‐sectional design (Baumgartner, Schiller, Rieskamp, et al., 2013; Wang, Li, Yin, Li, & Wei, 2016). By contrast, brain stimulation techniques, such as repetitive transcranial magnetic stimulation (rTMS), which interfere noninvasively with the activity of defined areas in the human cortex, allow researchers to draw causal conclusions about the behavioral impact of the stimulated brain region (Baumgartner, Schiller, Rieskamp, et al., 2013; Wang et al., 2016). Indeed, via rTMS, Baumgartner, Schiller, Rieskamp, et al. (2013) demonstrated that the right TPJ is causally involved in parochial punishment behavior. However, to the best of our knowledge, no other studies have used brain stimulation techniques to provide causal evidence about the impact of brain areas on intergroup bias. Thus, it is unclear whether the causal role of the right TPJ can be observed in other decision situations.

We, therefore, investigated the causal role of the right TPJ in intergroup bias in trust decisions by combining a behavioral economics task and rTMS. For the behavioral economics task, we modified a multi‐round trust game task, which is one of the most widely‐used games for assessing trust behavior (Berg, Dickhaut, & McCabe, 1995; Maurer, Chambon, Bourgeois‐Gironde, Leboyer, & Zalla, 2018). Because we aimed to examine the causal impact of the right TPJ on intergroup trust bias, we manipulated the counterparts' group membership (in‐group or out‐group) and reciprocity. We applied either neuronavigated inhibitory continuous theta burst stimulation (cTBS) (Huang, Edwards, Rounis, Bhatia, & Rothwell, 2005) or sham stimulation over the right TPJ before conducting the trust game task in healthy volunteers. Based on the accumulating evidence of intergroup trust bias from studies of group psychology (Balliet et al., 2014; De Dreu & Kret, 2016), we hypothesized that the participants would display significantly more trust toward in‐group members than they would toward out‐group members; that is, the degree of investment of the participants with in‐group members would be significantly greater than that dedicated to out‐group members in the trust game task. Furthermore, based on previous fMRI studies that showed that the right TPJ plays a vital role in the differentiation between in‐group and out‐group members in judgment and social behavior (Baumgartner et al., 2012; Falk et al., 2012; Wu et al., 2018), we hypothesized that the observed intergroup trust bias would be modulated by changes in the activation of this brain area. Regarding the direction of the effect, based on a previous rTMS study that demonstrated that the parochial punishment of social‐norm defectors was decreased after right TPJ inhibition (Baumgartner, Schiller, Rieskamp, et al., 2013), we hypothesized that the intergroup trust bias would be diminished after cTBS of the right TPJ.

## 2 MATERIALS AND METHODS

### 2.1 Participants

Twenty‐two healthy volunteers were enrolled in this study. We enrolled only male participants because of potential gender difference in intergroup trust bias (De Dreu & Kret, 2016; Gaertner & Insko, 2000; Wilson & Liu, 2003). All participants were right‐handed as assessed by the Edinburgh Handedness Inventory (Oldfield, 1971). The sample size was determined based on previous rTMS studies on decision‐making (Bardi, Six, & Brass, 2017; Baumgartner, Schiller, Rieskamp, et al., 2013; Krall et al., 2016). One participant was excluded from the analyses during data collection (see Supplementary Methods[Link] for details). Thus, data obtained from 21 participants were analyzed (aged 21–32 years, mean ± *SD* = 27.0 ± 3.7 years). No participants met the criteria for any psychiatric disorders according to the evaluation of an experienced psychiatrist using the Structured Clinical Interview for DSM‐IV Axis I Disorders (SCID I). No participants had a history of head trauma, serious medical or surgical illness, or substance abuse. The IQ was estimated as 106.0 ± 6.3 using a Japanese Version of the National Adult Reading Test short form (Matsuoka & Kim, 2006). Based on previous studies of decision‐making (Fujino et al., 2017, 2019; Pushkarskaya et al., 2015), we checked the participants' numeracy skills and understanding of numbers using a numeracy test, and all participants were judged to have the basic numeracy skills necessary to understand the task in this study (Supplementary Methods).

This study was approved by the institutional review board of Showa University Karasuyama Hospital and was conducted in accordance with the Code of Ethics of the World Medical Association. After providing a complete study description to all participants, written informed consent was obtained from all participants.

### 2.2 Design

The participants attended two experimental sessions where they received rTMS (real rTMS [cTBS] or sham rTMS) before engaging in the trust game task. To prevent carry‐over effects, the sessions were separated by at least 1 week, as reported previously (de Jesus et al., 2014). In addition, to control for order effects, the order of application of the stimulation condition (cTBS or sham rTMS) in each session was counterbalanced between the participants, based on the previous studies (Krall et al., 2016). Further details are described in the Supplementary Methods and Table S1.

### 2.3 rTMS

The rTMS procedure was performed using a Magstim Rapid^2^ system (Magstim Company, UK) with a 70‐mm figure‐of‐eight coil with a special air‐cooling system.

An inhibitory rTMS protocol (cTBS) was applied for the real rTMS (Huang et al., 2005). Bursts of three stimuli at 50 Hz were repeated with a frequency of 5 Hz for 40 s, resulting in a total of 600 pulses; the stimulation intensity was set to 80% of the active motor threshold. The active motor threshold was defined as the lowest pulse intensity required to elicit a motor‐evoked potential larger than 200 μV on more than 5 of 10 rounds from the contralateral first dorsal interosseous muscle while the subject was maintaining a contraction of ~20% maximum force (Huang et al., 2005; Soutschek, Ruff, Strombach, Kalenscher, & Tobler, 2016). For the sham rTMS, we implemented the same stimulation parameters used for the cTBS (location and rTMS pulse train properties) using a sham coil (Magstim Company).

**Figure 1:**
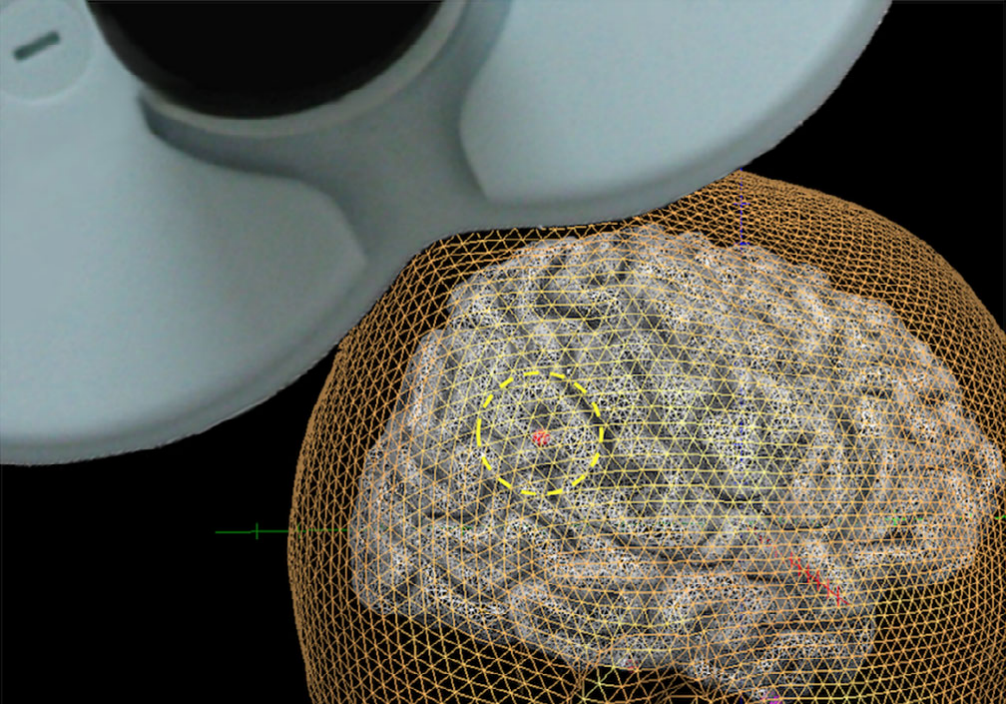
Stimulation targets. We localized the posterior part of the right temporoparietal junction (TPJ) at the Montreal Neurological Institute (MNI) coordinates (x = 54, y = −55, z = 26) obtained in the previous meta‐analysis study (Mars et al., 2012). The coordinates were transformed into the native space of each individual participant's scan (example presented in figure). By means of a neuronavigation system, the TMS coil was placed and kept during the stimulation in the scalp location underlying the targeted brain region

Prior to the experiment, structural T1‐weighted MRI scans of each participant were obtained on a 3 T Siemens Verio scanner with a 12‐channel phased‐array head coil. Three‐dimensional magnetization‐prepared rapid gradient‐echo (3D‐MPRAGE) sequences (TE = 3.06 ms, TR = 2000 ms, TI = 990 ms, FOV = 256 × 256 mm, matrix = 256 × 256, resolution = 1.0 × 1.0 × 1.0 mm^3^, and 208 total axial sections without intersection gaps) were used. We localized the right TPJ at the Montreal Neurological Institute (MNI) coordinates obtained in the previous meta‐analysis study (Mars et al., 2012). We used the coordinates of the posterior part of the right TPJ (x = 54, y = −55, z = 26), which have been reported to play a crucial role in social cognition, such as theory of mind and moral judgment (Donaldson, Rinehart, & Enticott, 2015; Mars et al., 2012) (Figure 1). The coordinates in the current study were close to the right TPJ (MNI: x = 57, y = −60, z = 30) stimulated in the previous rTMS study on parochial punishment (Baumgartner, Schiller, Rieskamp, et al., 2013). We transformed the right TPJ coordinates into the native space of each individual participant's scan using BrainVoyager QX TMS Neuronavigator software (Brain Innovation, Maastricht, Netherlands). A Zebris CMS20 ultrasound‐based system (Zebris Medical GmbH, Isny, Germany) was used for head and coil registration and monitoring.

### 2.4 Procedure and trust game task

We modified a multi‐round trust game task that has been used in previous studies (Berg et al., 1995; Hooper et al., 2019; Lemmers‐Jansen, Fett, Hanssen, Veltman, & Krabbendam, 2019; Maurer et al., 2018). In this study, all participants played the role of investors.

At the beginning of the first session, the participants completed a questionnaire regarding their social identities. Five categories were selected among those that are powerful sources of intergroup bias (hometown [Ben‐Ner et al., 2009; Dien, 2000], sports team loyalty [Balliet et al., 2014; Baumgartner, Schiller, Rieskamp, et al., 2013], political views [Falk et al., 2012; Wu et al., 2018], religion [Balliet et al., 2014; Hewstone et al., 2002], and music preference [Ben‐Ner et al., 2009; Tarrant, North, & Hargreaves, 2001]). Please see the Supplementary Methods for the details and rationale of the questionnaire. As a cover story, the participants were told that they would be divided into groups based on the answers to the questionnaire and would play with four anonymous partners in other rooms online. The participants were also told that two partners were selected from their group and the remaining two were from other group members. In reality, these partners were not real people, and the participants played against a computer that was programmed in advance.

**Figure 2:**
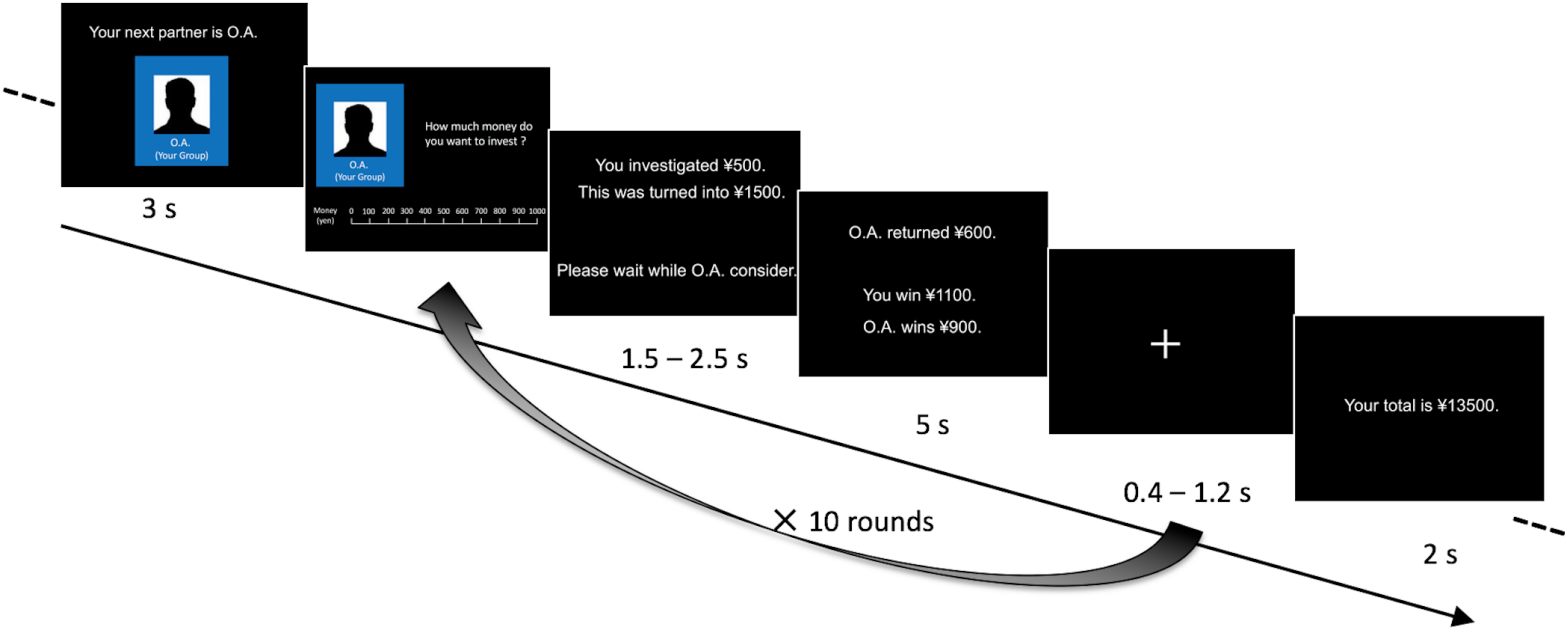
Trust game task. Following presentation of the initial name and group membership (in‐group or out‐group) of the counterpart, every participant (investor) played 10 consecutive rounds of the trust game with the same counterpart (trustee) before changing partners. In each round, the participants received ¥1,000, independent of previous actions. Then, the participants were instructed to choose an amount (between ¥0 and ¥1,000 [in increments of ¥100]) to give to their counterpart. The transferred amount was tripled, and the counterpart decided how much of the tripled amount to transfer back to the participant. After a short delay, the participant was informed of the counterpart's decision, and the amount earned in the round was shown

After the presentation of the initial name and group membership (in‐group or out‐group) of the counterpart, every participant (investor) played 10 consecutive rounds of the trust game with the same counterpart (trustee) before changing partners (Figure 2). In each round, the participants received ¥1,000 Japanese yen (~$10), independent of previous actions. Then, the participants were instructed to choose an amount (between ¥0 and ¥1,000 [in increments of ¥100]) to give to their counterpart. The transferred amount was tripled, and the counterpart decided how much of the tripled amount to transfer back to the investor. After a short delay, the participant was informed of the counterpart's decision, and the amounts earned in the round were shown. For example, if the amount being transferred by the investor is ¥X and the amount being transferred back by the counterpart is ¥Y, then the investor will receive ¥1,000 − ¥X + ¥Y, and the counterpart will receive ¥1,000 + ¥3X − ¥Y.

In the present version of the trust game task, we manipulated the counterpart's group membership (in‐group or out‐group) and reciprocity (cooperative or individualistic), thus giving rise to four different experimental conditions: in‐group/cooperative, in‐group/individualistic, out‐group/cooperative, and out‐group/individualistic. Unbeknownst to the participants, the two cooperative counterparts were programmed to play a strategy that returned higher sums than the participants initially invested (return ratios: 1/2, 8/15, 17/30, 3/5, 19/30, 2/3), and the two individualistic counterparts were designed to play a strategy in every round that never returned more money than the participants invested (return ratios: 1/6, 1/5, 7/30, 4/15, 3/10, 1/3) (see Table S2 for details). The presentation orders of the four virtual counterparts and their return ratios were randomized across participants.

We created two versions of the trust game task (versions A and B) to examine the stimulation condition effects (cTBS vs. sham rTMS) on the behavioral data. All participants performed both versions of the trust game task, and the order of the versions was counterbalanced across participants (see Table S1). The two versions of the trust game task were identical other than the initial names of the four virtual counterparts.

Based on the previous studies (Ben‐Ner et al., 2009; Bray, Shimojo, & O'Doherty, 2010; Fujino et al., 2017; Rosenberger, Ree, Eisenegger, & Sailer, 2018), the participants were told that their final participation fee would be determined depending on the predetermined ratio of earnings in the trust game task (at the end of the last session, we debriefed the participants on the purpose of the experiment and paid the maximum predefined participation fee [¥5,000 per session]).

All participants were quizzed regarding how well they understood the task (Supplementary Methods) and were corrected if there was any misunderstanding. Then, they practiced on a shorter version of the task at least once. Following this, the participants underwent either cTBS or sham rTMS to the right TPJ, before playing the trust game task immediately afterwards. The duration of the cTBS effects in disrupting activity in the stimulated brain region was expected to last at least 25–45 mins (Huang et al., 2005; Krall et al., 2016). Because the trust game task after the stimulation lasted ~10 min, we could be certain that the applied rTMS protocol reduced the excitability of the stimulated region during the full period of the task performance. This experiment was conducted using E‐Prime software (Psychology Software Tools, Inc., Pittsburgh, PA).

### 2.5 Statistical analysis

For the mean of the investment amounts and reaction time, we performed an analysis of variance (ANOVA) to examine stimulation condition effects (sham vs. cTBS), group membership effects (in‐group vs. out‐group), and reciprocity effects (cooperative vs. individualistic) and the interaction of these factors. The statistical analyses were performed using SPSS v.24 (IBM Corp., Armonk, NY). Results were considered statistically significant at *p* < .05 (two‐tailed). The thresholds of statistical significance of post hoc *t* tests were adjusted by the Bonferroni correction.

## 3 RESULTS

### 3.1 Right TPJ in intergroup trust bias

**Figure 3:**
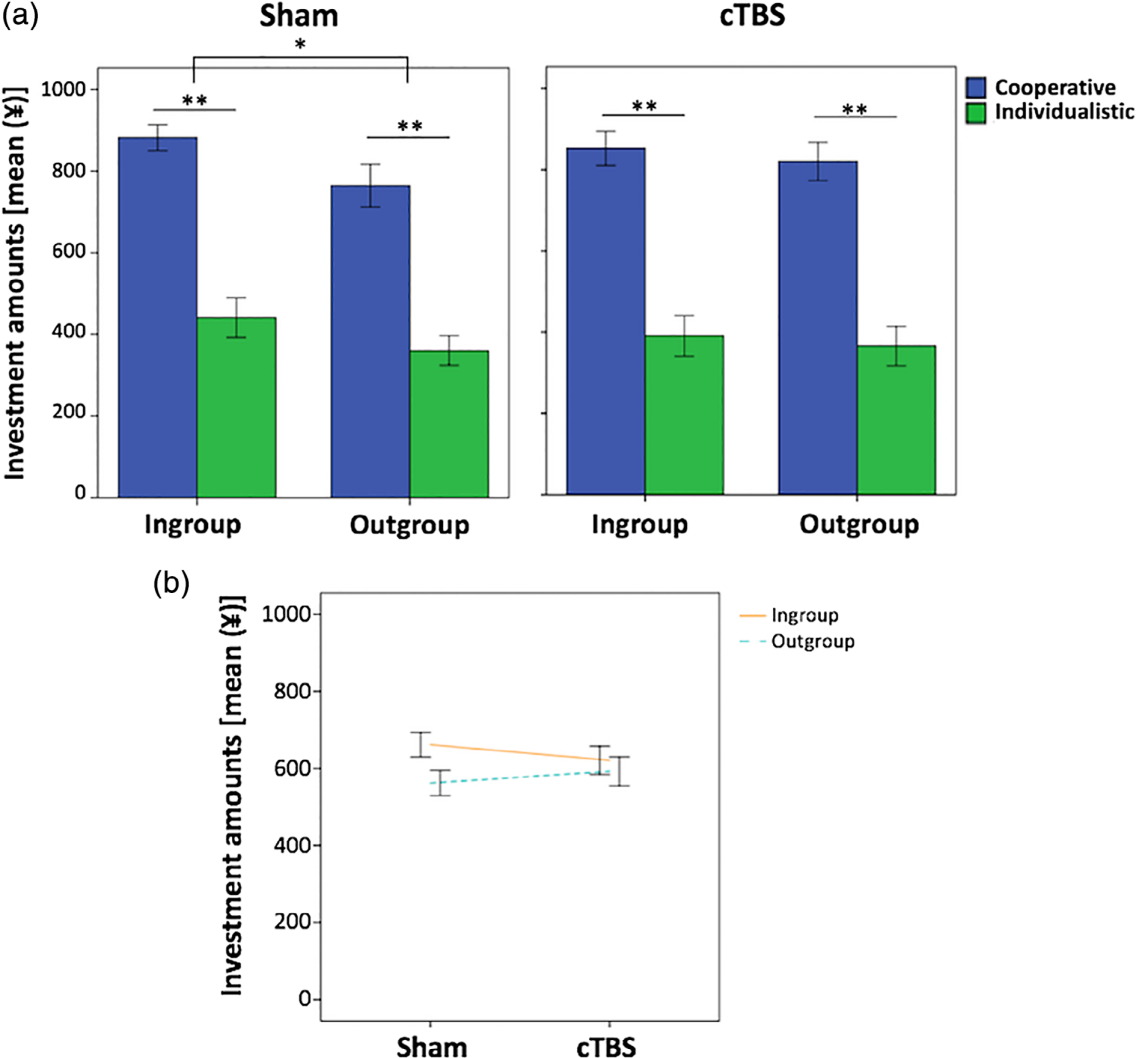
Right temporoparietal junction (TPJ) in intergroup trust bias. (a) Amounts invested in the trust game task after sham stimulation and continuous theta burst stimulation (cTBS). *p < .05, **p < .01. (b) The 2 (stimulation condition [sham vs. cTBS]) × 2 (group membership [in‐group vs. out‐group]) × 2 (reciprocity [cooperative vs. individualistic]) repeated‐measures analysis of variance revealed the presence of a stimulation condition × group membership interaction effect (F [1, 20] = 4.87, *p* = .039). The error bars indicate ± *SE*

The effects of stimulation on the participants' investments in the trust game task were analyzed in a 2 (stimulation condition [sham vs. cTBS]) × 2 (group membership [in‐group vs. out‐group]) × 2 (reciprocity [cooperative vs. individualistic]) repeated‐measures ANOVA. The results showed that the main effect of the stimulation condition was not significant (*F* [1, 20] = 0.03, *p* = .87, Table 1), meaning that there was no statistical difference in the overall investment amounts between the sham stimulation and cTBS (Figure 3a). However, we did find a significant main effect of group membership (*F* [1, 20] = 7.24, *p* = .014), indicating that the amounts invested in in‐group members were significantly higher than those in out‐group members (Figure 3a). In addition, a significant stimulation condition × group membership interaction (*F* [1, 20] = 4.87, *p* = .039) was observed. After the sham stimulation, the amounts invested in in‐group members were significantly higher than those invested in out‐group members (*p* = 0.012, Figure 3b). However, there was no significant difference in the amounts invested between in‐group and out‐group members following cTBS to the right TPJ (*p* = .14, Figure 3b). There were no significant differences in the amounts invested toward in‐group members or out‐group members between the sham stimulation and cTBS (*p* = .23 and 0.41, respectively). The findings were not significantly affected by the order of stimulation (Supplementary Results).

**Table 1:** Results of the ANOVA for investment amounts and reaction time in the trust game task

	Investment amounts		Reaction time	
	*F* value	*p* value	*F* value	*p* value
Stimulation condition	0.03	.87	0.01	.92
Group membership	7.24	.014*	0.65	.43
Reciprocity	134.70	< .01**	2.40	.14
Stimulation condition × group membership	4.87	.039*	0.64	.43
Stimulation condition × reciprocity	0.24	.63	2.71	.12
Group membership × reciprocity	0.34	.56	2.92	.10
Stimulation condition × group membership × reciprocity	0.21	.65	0.04	.85

*Abbreviation*: ANOVA, analysis of variance.

*p < .05; **p < .01.

**Figure 4:**
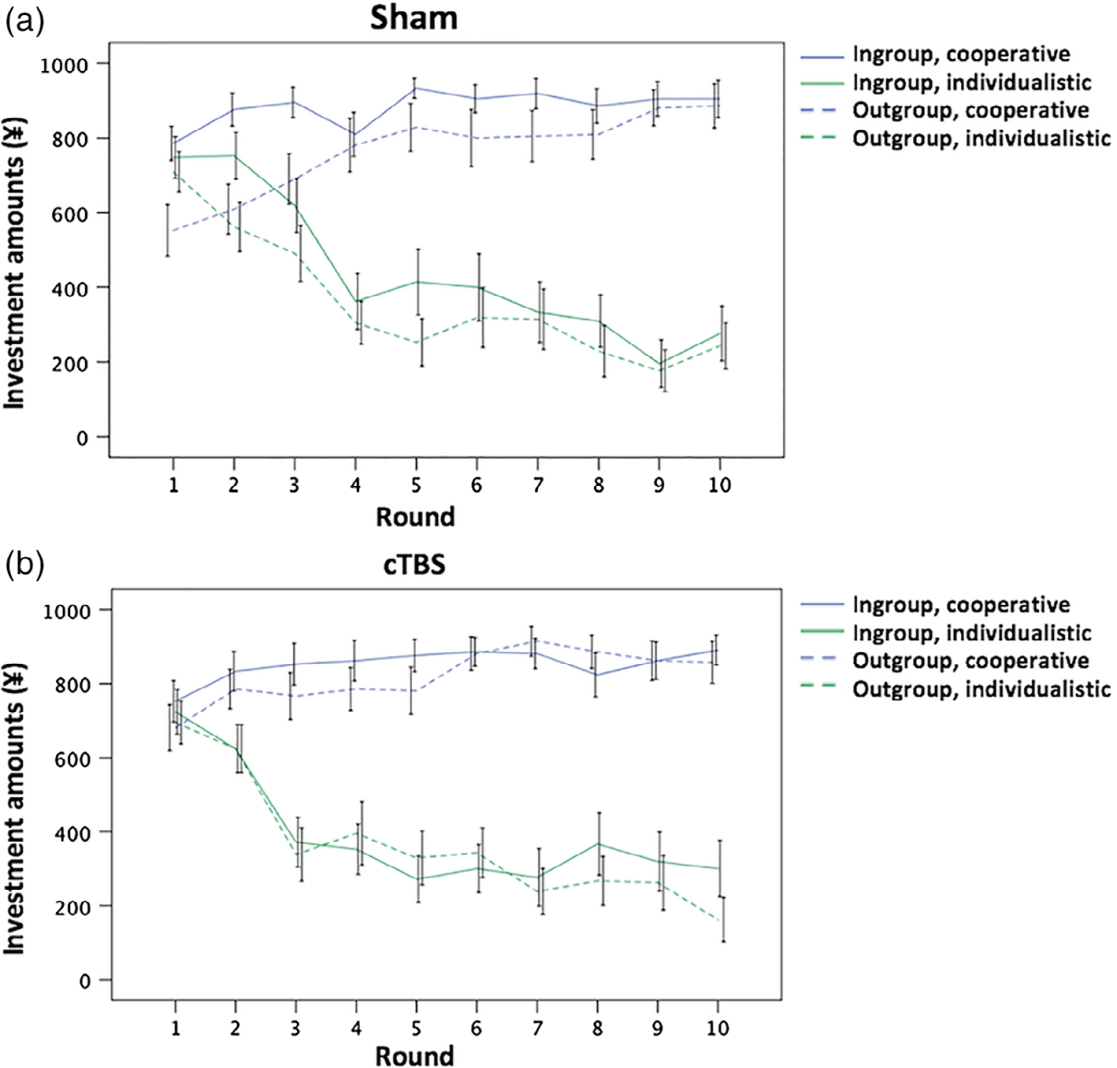
Round‐by‐round investments of the participants. (a) Amounts invested by the participants after the sham stimulation. (b) Amounts invested by the participants after continuous theta burst stimulation (cTBS). The error bars indicate ± *SE*

A significant main effect of reciprocity was also observed (*F* [1, 20] = 134.70, *p* < .01), indicating that the participants invested more in cooperative partners compared to individualistic partners (Figure 3a). Neither the stimulation condition × reciprocity (*F* [1, 20] = 0.24, *p* = .63), nor the group membership × reciprocity (*F* [1, 20] = 0.34, *p* = .56), nor the three‐way stimulation condition × group membership × reciprocity (*F* [1, 20] = 0.21, *p* = .65) interactions were significant. Each of the amounts invested during the 10 rounds of the four experimental conditions are shown in Figure 4.

We also performed a 2 × 2 × 2 repeated‐measures ANOVA for reaction time based on stimulation condition (sham vs. cTBS) × group membership (in‐group vs. out‐group) × reciprocity (cooperative vs. individualistic). There were no significant main effects of stimulation condition, group membership, and reciprocity. Neither the stimulation condition × reciprocity, nor the stimulation condition × group membership, nor the group membership × reciprocity, nor the three‐way stimulation condition × group membership × reciprocity interactions were significant (Table 1).

### 3.2 Effect of round on investments

**Figure 5:**
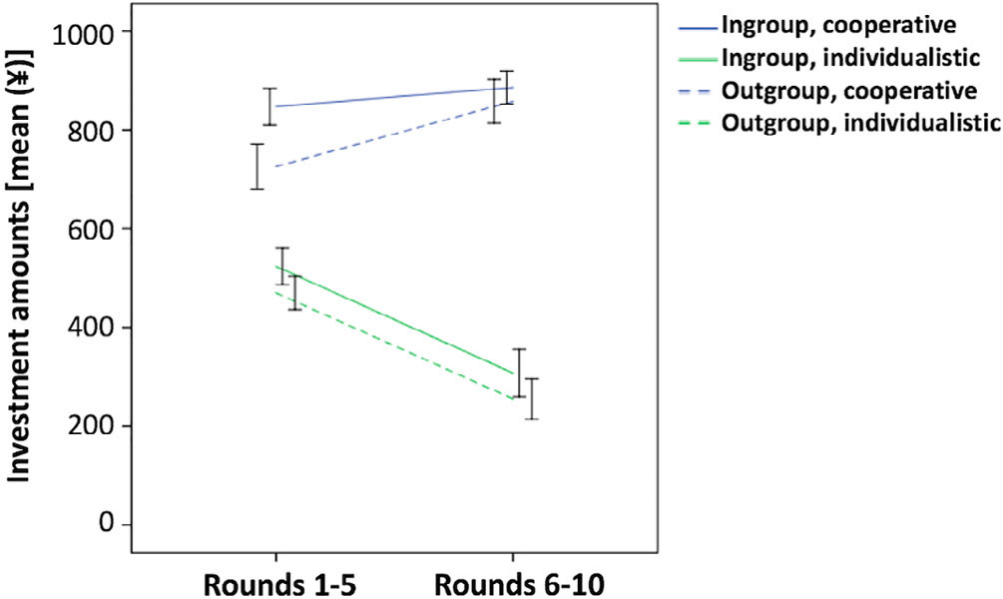
Effect of round on investments. The results of the 2 (stimulation condition [sham vs. cTBS]) × 2 (group membership [in‐group vs. out‐group]) × 2 (reciprocity [cooperative vs. individualistic]) × 2 (round [former half vs. latter half]) repeated‐measures analysis of variance revealed the presence of a significant three‐way group membership × reciprocity × round interaction (F [1, 20] = 6.21, *p* = .022)

To explore the effect of round on the participants' investment behavior, we dichotomized the 10 rounds into the former half (rounds 1–5) and the latter half (rounds 6–10). Subsequently, we ran an additional ANOVA for the mean of the investment amounts with including the “round” factor in the analysis; that is, a 2 (stimulation condition [sham vs. cTBS]) × 2 (group membership [in‐group vs. out‐group]) × 2 (reciprocity [cooperative vs. individualistic]) × 2 (round [former half vs. latter half]) repeated‐measures ANOVA was performed. We detected a significant main effect of the round factor (*F* [1, 20] = 8.44, *p* < .01) as well as significant reciprocity × round (*F* [1, 20] = 60.81, *p* < .01) and three‐way group membership × reciprocity × round (*F* [1, 20] = 6.21, *p* = .022) interactions (Figure 5). The stimulation condition × round, the group membership × round, the three‐way stimulation condition × group membership × round, the three‐way stimulation condition × reciprocity × round, and the four‐way stimulation condition × group membership × reciprocity × round interactions were not significant (all, *p* > .09, Table S3).

The three‐way group membership × reciprocity × round interaction (Figure 5) was further investigated via two‐way ANOVA using the group membership and reciprocity factors separately for each half of the rounds. In the former half of the rounds, main effects of group membership (*F* [1, 20] = 9.11, *p* < .01) and reciprocity (*F* [1, 20] = 103.77, *p* < .01) were detected. In addition, a significant group membership × reciprocity interaction (*F* [1, 20] = 4.54, *p* = .046) was observed. Decomposing the group membership × reciprocity interaction, pair‐wise comparisons revealed that the amounts invested toward in‐group members were significantly higher than those invested toward out‐group members in the cooperative partners condition (*p* < .01), whereas this difference was not significant in the individualistic partners condition (*p* = .12). The investment amounts toward cooperative partners were significantly higher than those toward individualistic partners in both in‐group and out‐group conditions (both, *p* < .01). As for the latter half of the rounds, we also found a significant main effect of reciprocity (*F* [1, 20] = 124.08, *p* < .01). However, neither the main effect of group membership (*F* [1, 20] = 2.52, *p* = .13) nor the group membership × reciprocity interaction (*F* [1, 20] = 0.25, *p* = .63) was significant. As shown in Figure 4, these observations were considered mainly because investments toward cooperative partners in in‐group members reached a ceiling rapidly during the task, whereas the amounts invested in the remaining three experimental conditions increased or decreased along with the number of rounds.

## 4 DISCUSSION

To the best of our knowledge, this is the first rTMS study to investigate the causal role of the right TPJ in intergroup bias in trust decisions. As expected, the participants invested significantly more in in‐group members compared with out‐group members in our task, which indicates that the participants' decisions were influenced by group membership. The results were in line with the previous studies of group psychology (Balliet et al., 2014; De Dreu & Kret, 2016). Notably, a significant stimulation condition × group membership interaction was observed. After the sham stimulation, the amounts invested in in‐group members were significantly higher than those invested in out‐group members. However, after cTBS to the right TPJ, this difference was not observed. These results suggest that the intergroup trust bias is diminished after the inhibition of the right TPJ.

The TPJ is involved in multiple cognitive functions (Alkire, Levitas, Warnell, & Redcay, 2018; Baumgartner, Dahinden, Gianotti, & Knoch, 2019; Donaldson et al., 2015; Fujino, Yamasaki, et al., 2014; Mars et al., 2012; Soutschek et al., 2016). In particular, the right TPJ plays a key role in social cognition, such as perspective taking (Krall et al., 2016; Schurz, Tholen, Perner, Mars, & Sallet, 2017; Tei et al., 2014), moral decision‐making (Bitsch, Berger, Nagels, Falkenberg, & Straube, 2018; Chen, Decety, Huang, Chen, & Cheng, 2016; Tei et al., 2017, 2019; Young, Camprodon, Hauser, Pascual‐Leone, & Saxe, 2010), and strategic social behavior (Hampton, Bossaerts, & O'Doherty, 2008; Hill et al., 2017). Significantly, previous fMRI studies have shown that the right TPJ plays a vital role in differentiating between in‐group and out‐group members in judgments and behavior (Baumgartner et al., 2012; Falk et al., 2012; Wu et al., 2018). In addition, a previous rTMS study have demonstrated that parochial punishment of social‐norm defectors was decreased following right TPJ inhibition (Baumgartner, Schiller, Rieskamp, et al., 2013). The authors proposed that the right TPJ is causally involved in parochialism in intergroup conflict (Baumgartner, Schiller, Rieskamp, et al., 2013). Taken together, our results are in line with these previous experimental findings and demonstrate that the causal role of the right TPJ in intergroup bias can also be observed in the context of the trust decisions.

Previous studies have shown that certain brain areas located near the TPJ, such as the inferior parietal cortex, play roles in attention and the processing of more general task performance (Baumgartner, Schiller, Rieskamp, et al., 2013; Corbetta, Kincade, Ollinger, McAvoy, & Shulman, 2000; Van Overwalle, 2011). Therefore, one may ask whether the current findings are rather unspecific and caused by diminished attention or generally diminished task performance. However, this is unlikely because we found a significant stimulation condition × group membership interaction, whereas the main effect of stimulation condition, the stimulation condition × reciprocity, and the three‐way stimulation condition × group membership × reciprocity interactions were not significant. Such specific findings would be difficult to explain based on unspecific attentional or cognitive processes. In addition, no effects of stimulation condition on reaction times were detected. Repeated‐measures ANOVA for reaction time based on stimulation condition × group membership × reciprocity revealed neither a main effect of stimulation condition nor interaction effects of stimulation condition with one or both of the other factors. These results are also inconsistent with the assumption that our findings can be attributed to an unspecific effect of diminished attention or generally diminished task performance because such an effect may lead to slower reaction times (Baumgartner, Schiller, Rieskamp, et al., 2013; Tzambazis & Stough, 2000).

Regarding the effect of round on the investments made by the participants, we found a significant three‐way group membership × reciprocity × round interaction. In the former half of the rounds, we detected main effects of group membership and reciprocity and a significant group membership × reciprocity interaction. However, in the latter half of the rounds, the main effect of group membership and the significant group membership × reciprocity interaction disappeared. As shown in Figure 4, these observations can be explained mostly by the fact that investments toward cooperative partners in in‐group members reached a ceiling rapidly during the task, whereas the amounts invested in the remaining three experimental conditions increased or decreased along with the number of rounds. Previous studies have shown that intergroup discrimination is mostly caused by in‐group favoritism (behavior that benefits one's in‐group) and rarely by out‐group derogation (behavior that aggresses and harms rivaling out‐groups) (Balliet et al., 2014; De Dreu & Kret, 2016). In addition, developmental research has shown that in‐group love develops earlier in childhood compared with out‐group hate (Balliet et al., 2014; Buttelmann & Böhm, 2014; Fehr, Glätzle‐Rützler, & Sutter, 2013). In line with this notion, the investments made by our participants toward cooperative partners in in‐group members may have increased immediately and intensively. This speculation should be examined in future studies using various scenarios and a wide range of rounds.

Although extreme intergroup bias can lead to severe outcomes, such as excessive competition, discrimination, and violent protest, this tendency improves group functioning and enables the individual to fit into a group (Balliet et al., 2014; Baumgartner, Schiller, Rieskamp, et al., 2013). Thus, the ability to distinguish the behaviors of in‐group and out‐group members is considered to have developed through evolution (Balliet et al., 2014; Baumgartner, Schiller, Rieskamp, et al., 2013). Patients with various psychiatric disorders, such as ASD and schizophrenia, have a reduced capacity to read and adapt to prevalent group norms and practices and often fail to trust and cooperate even with individuals who are close to them (De Dreu & Kret, 2016; Fujino, Takahashi, et al., 2014; King‐Casas et al., 2008; Tei, Fujino, et al., 2019). Therefore, these patients have difficulty forming and maintaining social bonds and suffer from social exclusion and isolation (De Dreu & Kret, 2016). Our findings may offer clues for studying social cognitive impairments in psychiatric disorders in terms of group psychology. Significantly, dysfunction of the right TPJ has been reported repeatedly in many psychiatric disorders (Lee, Quintana, Nori, & Green, 2011; Philip et al., 2012; von dem Hagen, Stoyanova, Baron‐Cohen, & Calder, 2012). For example, previous studies demonstrated the existence of a link between aberrant right TPJ function during mentalizing tasks and social impairments in ASD (Donaldson et al., 2015; Lombardo, Chakrabarti, Bullmore, Baron‐Cohen, & Consortium, 2011). The examination of the potential effects of right TPJ modulation in psychiatric disorders such as ASD will be an important avenue of research in the future.

This study has several limitations. First, the sample size was small. Thus, our findings should be interpreted cautiously. For example, we did not find significant differences in the amounts invested toward in‐group members or out‐group members between the sham stimulation and cTBS. Such null findings should be considered in the context of a low power for detecting significant differences. Second, we did not perform the experiment using a real social group; rather, we created a situational setting for the participants in which they were facing their counterparts from the in‐group/out‐group using a cover story. In addition, because the actual expected earnings in the trust game task were relatively expensive for the winning rewards of the experiment, the participants were told that their final participation fee would be determined depending on the predetermined ratio of earnings in the task; at the end of the last session, we debriefed them on the purpose of the experiment and paid the maximum predefined participation fee. Nevertheless, the post‐task interview confirmed that the participants were led to believe that they were playing with real people and that their decisions had real consequences. Furthermore, none of the participants showed illogical behavior (invested more money toward individualistic partners than toward cooperative partners) or who were more than 2 SD below the mean regarding reaction time [an extremely fast reaction time implies poor decision quality (e.g., Greenwald, Nosek, & Banaji, 2003; Tei et al., 2018)], which supports our contention that all the participants made substantial efforts to tackle the trust game task. Therefore, we believe that our findings are useful for understanding the role of the right TPJ in intergroup bias in trust decisions. Third, our sample consisted of only males. Previous studies have shown that women identify with their in‐group more strongly than men (Wilson & Liu, 2003) and that women show in‐group favoritism regardless of whether they are dependent on the in‐group, whereas men show this tendency when they depend on in‐group members for outcomes (Gaertner & Insko, 2000). Thus, our present findings may not be generalized to female subjects. Future studies should recruit female subjects and discuss gender effect using adequate statistical analysis. Notwithstanding these limitations, the current results extend previous findings by showing that the inhibition of the right TPJ leads to reduced intergroup bias in trust decision situations.

## 5 CONCLUSIONS

By combining a behavioral economics task and rTMS, we demonstrate that the right TPJ is causally involved in intergroup bias in trust decisions. Our findings contribute to a better understanding of the mechanisms of human social behavior.

## CONFLICT OF INTERESTS

All authors declare that they have no conflicts of interest.

## Supporting information


**Appendix S1:** Supporting information

## Data Availability

The data that support the findings of this study are available from the corresponding author upon reasonable request.
