# Supplementary material for: Role of the right temporoparietal junction in intergroup bias in trust decisions
Source: Hum Brain Mapp. 2019 Dec 19;41(6):1677–88. doi: 10.1002/hbm.24903 (PMC7268017; doi:10.1002/hbm.24903)
Supplement: Supplementary file 1 — Appendix S1: Supporting information [file HBM-41-1677-s001.docx]

**Supplementary Materials**

**Title:** Role of the right temporoparietal junction in intergroup bias in trust decisions

**Short Title:** Temporoparietal junction in intergroup bias

Junya Fujino^1,2,†^, Shisei Tei^1,2,3,4,†^, Takashi Itahashi^1^, Yuta Y. Aoki^1^, Haruhisa Ohta^1,5^, Manabu Kubota^1,2,6^, Ryu-ichiro Hashimoto^1,7^, Hidehiko Takahashi^1,2,8^, Nobumasa Kato^1^, Motoaki Nakamura^1,9^

^1^Medical Institute of Developmental Disabilities Research, Showa University, 6-11-11 Kita-karasuyama, Setagaya-ku, Tokyo, Japan

^2^Department of Psychiatry, Graduate School of Medicine, Kyoto University, 54 Shogoin-Kawaracho, Sakyo-ku, Kyoto, Japan

^3^Institute of Applied Brain Sciences, Waseda University, 2-579-15 Mikajima, Tokorozawa, Saitama, Japan

^4^School of Human and Social Sciences, Tokyo International University, 2509 Matoba, Kawagoe, Saitama, Japan

^5^Department of Psychiatry, School of Medicine, Showa University, 6-11-11 Kita-karasuyama, Setagaya-ku, Tokyo, Japan

^6^Department of Functional Brain Imaging, National Institute of Radiological Sciences, National Institutes for Quantum and Radiological Science and Technology, 4-9-1 Anagawa, Inage-ku, Chiba, Japan

^7^Department of Language Sciences, Graduate School of Humanities, Tokyo Metropolitan University, 1-1 Minami-Osawa, Hachioji-shi, Tokyo, Japan

^8^Department of Psychiatry and Behavioral Sciences, Graduate School of Medical and Dental Sciences, Tokyo Medical and Dental University, 1-5-45 Yushima, Bunkyo-ku, Tokyo, Japan

^9^Kanagawa Psychiatric Center, 2-5-1 Serigaya, Yokohama, Kanagawa, Japan

^†^These authors contributed equally to this work.

**Correspondence:** Junya Fujino, M.D., Ph.D., Motoaki Nakamura, M.D., Ph.D.

Medical Institute of Developmental Disabilities Research, Showa University, 6-11-11 Kita-karasuyama, Setagaya-ku, Tokyo, 157-8577, Japan

Tel: +81-3-5315-9357, Fax: +81-3-5315-9358,

E-mail: [fujinoj@med.showa-u.ac.jp](mailto:fujinoj@med.showa-u.ac.jp) (J.F.), motoaki@motoaki.com (M.N.)

**Supplementary Methods**

***Exclusion of the participants***

One participant was excluded from the analyses. Because his total investment amounts in some of the four experimental conditions (cTBS or sham rTMS) were too low (nearly ¥0), he almost never had the opportunity to know the reciprocity of the partners in those conditions. Thus, his behavioral data were not considered an adequate reflection of the reciprocity factor of our trust game task. More precisely, his total amounts invested in the outgroup/cooperative condition after cTBS were ¥100 (possible max. amounts, ¥10000), and those in the ingroup/individualistic and outgroup/individualistic conditions after sham rTMS were ¥300 and ¥200, respectively. The other participants’ total amounts invested in each of the four experimental conditions (cTBS or sham rTMS) were much higher (all $\geq$ ¥ 1000).

***Design***

The participants attended two experimental sessions where they received rTMS [real rTMS (cTBS) or sham rTMS] before engaging in the trust game task. After completing the trust game task, all participants performed another decision-making task, which will be reported elsewhere. The sessions were separated by at least one week to prevent carry-over effects. The order of the stimulation condition (cTBS or sham rTMS) applied at each session was counterbalanced between participants (10 participants received cTBS and 11 participants received sham rTMS on the first session, Table S1).

***Numeracy test***

1. You have ¥1000. How much money will you have if you use ¥300?

_________________ (correct answer [¥700])

1. You have ¥600. How much money will you have if you receive ¥200?

_________________ (correct answer [¥800])

1. How much is three times ¥400?

________________ (correct answer [¥1200])

1. Which of the following is the highest?
2. ¥300 + ¥500
3. ¥1100 + ¥100
4. ¥700 + ¥800

________________ (correct answer [c])

1. Which of the following is the highest?
2. ¥1300 − ¥500
3. ¥1500 − ¥800
4. ¥800 − ¥200

________________ (correct answer [a])

***Questionnaire for grouping***

1. Where is your hometown?

______________________________________________________

1. Which baseball team do you cheer for?

_____________________________________________________

1. Which political party do you support?

______________________________________________________

1. Which religion do you believe in?

______________________________________________________

1. What music do you like?

______________________________________________________

***Rationale of the questionnaire***

We recruited healthy volunteers from the general population via advertisements and acquaintances. Thus, our participants were considered to have different interests and habits. In this setting, and to increase credibility, the questionnaire included a series of categories that covered the interests/habits of all of the participants of this study, based on the previous studies (Akerlof & Kranton, 2000; Ben-Ner et al., 2009) (in fact, all participants provided detailed answers to at least two categories). Five categories were selected among those that are powerful sources of intergroup bias [hometown (Ben-Ner et al., 2009; Dien, 2000), sports team loyalty (Balliet et al., 2014; Baumgartner et al., 2013), political views (Falk et al., 2012; Wu et al., 2018), religion (Balliet et al., 2014; Hewstone et al., 2002), and music preference (Ben-Ner et al., 2009; Tarrant et al., 2001)].

***Quiz for the trust game task***

1. You invest ¥800. Then, how much money do you have at this moment?


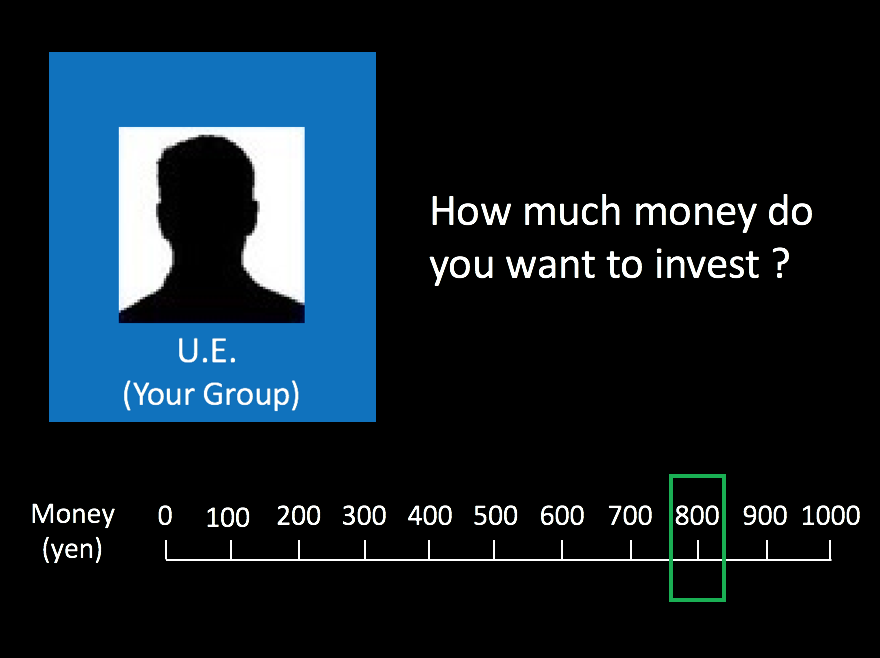


(Correct answer, \200)

2. How much money did U.E. receive?


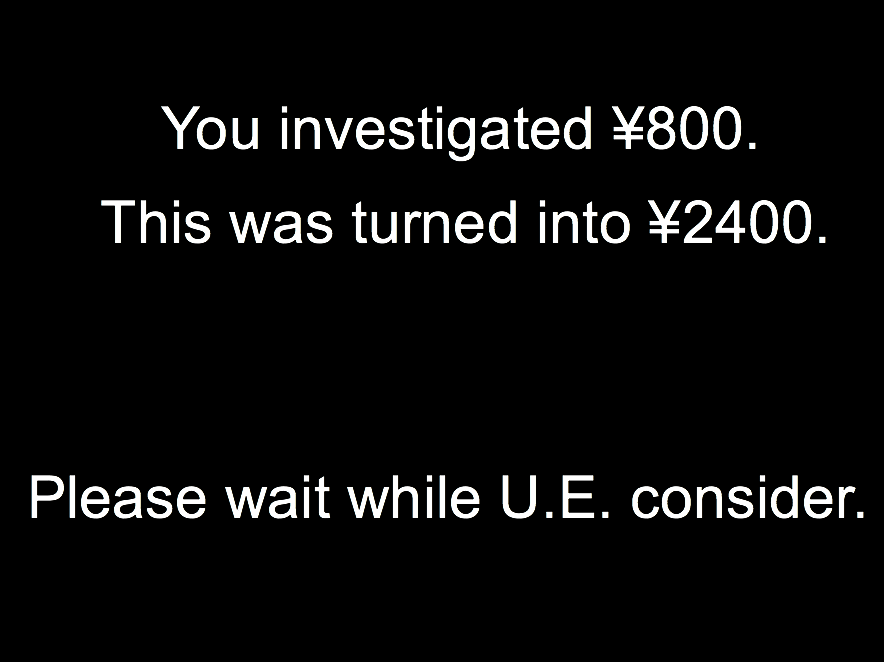


(Correct answer, ¥2400)

3. U.E. returned ¥1000. How much money did you gain in this round?


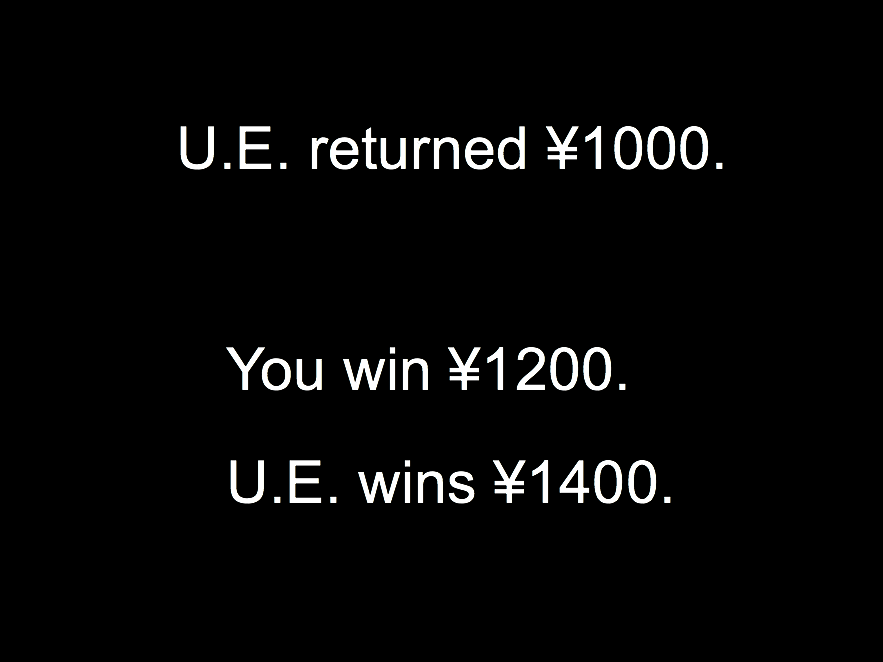


(Correct answer, ¥1200)

4. How much money did U.E. gain in this round?


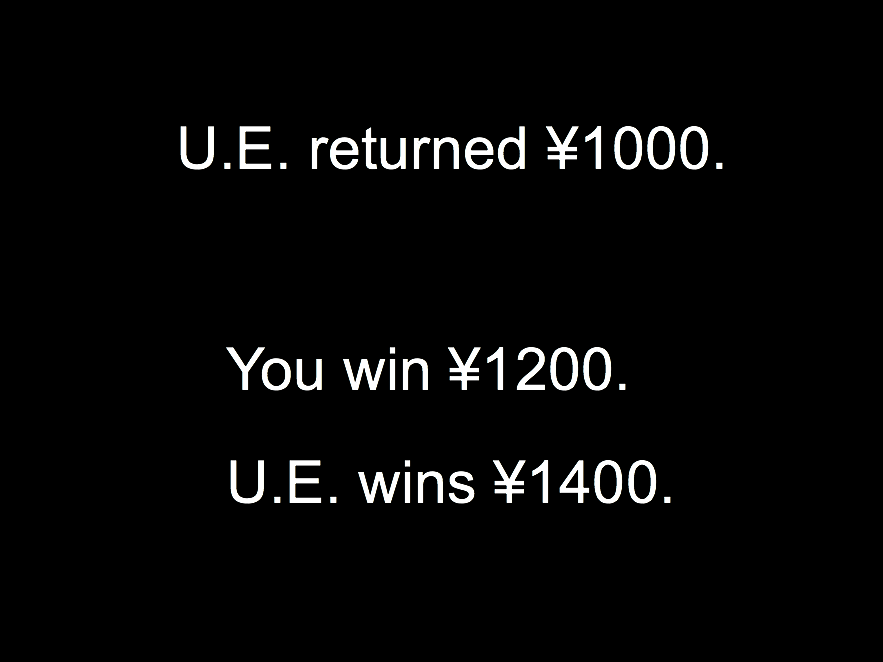


(Correct answer, ¥1400)

**Supplementary Results**

***Analysis of the effect of the order of stimulation***

To test the effect of the order of stimulation on our findings, we performed a 2 × 2 × 2 ANOVA using order (sham in the 1^st^ session and cTBS in the 2^nd^ session vs. cTBS in the 1^st^ session and sham in the 2^nd^ session) as the between-subject factor and stimulation condition (sham vs. cTBS) and group membership (ingroup vs. outgroup) as the within-subject factors for the investment amounts in the trust game task. This analysis revealed the presence of a significant main effect of group membership [*F* (1, 19) = 7.47, *p* = 0.013] and a stimulation condition × group membership interaction [*F* (1, 19) = 4.68, *p* = 0.043]. However, the main effect of order, the order × stimulation condition, the order × group membership, and the three-way order × stimulation condition × group membership interactions were not significant (all, *p* > 0.10). These results confirmed that the order of stimulation did not affect our conclusions.

**Supplementary References**

Akerlof, G. A., & Kranton, R. E. (2000). Economics and identity. The quarterly journal of economics, 115(3), 715-753.

Ben-Ner, A., McCall, B. P., Stephane, M., & Wang, H. (2009). Identity and in-group/out-group differentiation in work and giving behaviors: Experimental evidence. Journal of Economic Behavior & Organization, 72(1), 153-170.

Dien, D. S.-f. (2000). The evolving nature of self-identity across four levels of history. Human Development, 43(1), 1-18.

Balliet, D., Wu, J., & De Dreu, C. K. (2014). Ingroup favoritism in cooperation: A meta-analysis. Psychological Bulletin, 140(6), 1556-1581.

Baumgartner, T., Schiller, B., Rieskamp, J., Gianotti, L. R., & Knoch, D. (2013). Diminishing parochialism in intergroup conflict by disrupting the right temporo-parietal junction. Social Cognitive and Affective Neuroscience, 9(5), 653-660.

Falk, E. B., Spunt, R. P., & Lieberman, M. D. (2012). Ascribing beliefs to ingroup and outgroup political candidates: neural correlates of perspective-taking, issue importance and days until the election. Philosophical Transactions of the Royal Society B: Biological Sciences, 367(1589), 731-743.

Hewstone, M., Rubin, M., & Willis, H. (2002). Intergroup bias. Annual Review of Psychology, 53(1), 575-604.

Tarrant, M., North, A. C., & Hargreaves, D. J. (2001). Social categorization, self-esteem, and the estimated musical preferences of male adolescents. The Journal of Social Psychology, 141(5), 565-581.

Wu, C.-T., Fan, Y.-T., Du, Y.-R., Yang, T.-T., Liu, H.-L., Yen, N.-S., … Hsung, R.-M. (2018) How do acquired political identities influence our neural processing toward others within the context of a trust game? Frontiers in Human Neuroscience, 12, 23.

**Table S1. Order of the stimulation condition and trust game task**

| Participant | Stimulation condition | |  | Trust game task | |
| --- | --- | --- | --- | --- | --- |
|  | Session 1 | Session 2 |  | Session 1 | Session 2 |
| 1 | sham | cTBS |  | A | B |
| 2 | cTBS | sham |  | A | B |
| 3 | sham | cTBS |  | B | A |
| 4 | cTBS | sham |  | B | A |
| 5 | sham | cTBS |  | A | B |
| 6 | cTBS | sham |  | A | B |
| 7 | sham | cTBS |  | B | A |
| 8 | cTBS | sham |  | B | A |
| 9 | sham | cTBS |  | A | B |
| 10 | cTBS | sham |  | A | B |
| 11 | sham | cTBS |  | B | A |
| 12 | cTBS | sham |  | B | A |
| 13 | sham | cTBS |  | A | B |
| 14 | cTBS | sham |  | A | B |
| 15 | sham | cTBS |  | B | A |
| 16 (excluded) | cTBS | sham |  | B | A |
| 17 | sham | cTBS |  | A | B |
| 18 | cTBS | sham |  | A | B |
| 19 | sham | cTBS |  | B | A |
| 20 | cTBS | sham |  | B | A |
| 21 | sham | cTBS |  | A | B |
| 22 | cTBS | sham |  | A | B |

Abbreviations: cTBS = continuous theta burst stimulation

**Table S2. Return ratio of the counterparts (trustees) in the trust game task**

| Ingroup | |  | Outgroup | |
| --- | --- | --- | --- | --- |
| Cooperative | Individualistic |  | Cooperative | Individualistic |
| 1/2 | 1/6 |  | 1/2 | 1/6 |
| 8/15 | 1/5 |  | 8/15 | 1/5 |
| 17/30 | 7/30 |  | 17/30 | 7/30 |
| 3/5 | 4/15 |  | 3/5 | 4/15 |
| 19/30 | 3/10 |  | 19/30 | 3/10 |
| 2/3 | 1/3 |  | 2/3 | 1/3 |
| 1/2 | 1/6 |  | 1/2 | 1/6 |
| 17/30 | 7/30 |  | 17/30 | 7/30 |
| 3/5 | 4/15 |  | 3/5 | 4/15 |
| 2/3 | 1/3 |  | 2/3 | 1/3 |

Every participant (investor) played 10 consecutive rounds of the trust game with the same counterpart before changing partners. The presentation orders of the four virtual counterparts and their return ratios were randomized across participants.

**Table S3. Results of the additional ANOVA of investment amounts including the “round” factor in the trust game task**

|  | Investment amounts | |
| --- | --- | --- |
|  | *F* value | *p* value |
| Stimulation condition | 0.03 | 0.87 |
| Group membership | 7.24 | 0.014* |
| Reciprocity | 134.70 | < 0.01** |
| Round | 8.44 | < 0.01** |
| Stimulation condition × Group membership | 4.87 | 0.039* |
| Stimulation condition × Reciprocity | 0.24 | 0.63 |
| Stimulation condition× Round | 0.40 | 0.53 |
| Group membership × Reciprocity | 0.34 | 0.56 |
| Group membership ×Round | 3.15 | 0.09 |
| Reciprocity ×Round | 60.81 | < 0.01** |
| Stimulation condition × Group membership × Reciprocity | 0.21 | 0.65 |
| Stimulation condition × Group membership × Round | 2.41 | 0.14 |
| Stimulation condition ×Reciprocity × Round | 1.91 | 0.18 |
| Group membership ×Reciprocity × Round | 6.21 | 0.022 |
| Stimulation condition × Group membership × Reciprocity× Round | 2.28 | 0.15 |

**p* < 0.05, ***p* < 0.01

Abbreviations: ANOVA = analysis of variance
